# Efficacy of transcutaneous auricular vagus nerve stimulation in treating patients with post-stroke motor disorders: a prospective study

**DOI:** 10.3389/fneur.2026.1711146

**Published:** 2026-03-03

**Authors:** Ruiling Xue, Jingxi Ma

**Affiliations:** 1 Chongqing Medical University, Chongqing, China; 2 Department of Rehabilitation, Chongqing General Hospital, Chongqing, China; 3 Department of Neurology, Chongqing General Hospital, Chongqing, China

**Keywords:** motor disorders, neural plasticity, rehabilitation training, stroke, transcutaneous auricular vagus nerve stimulation

## Abstract

**Background:**

Although traditional rehabilitation training can partially improve motor function in patients with post-stroke motor disorders, its impact on neural plasticity remains limited. Transcutaneous auricular vagus nerve stimulation (taVNS), a non-invasive method targeting the auricular branch of the vagus nerve, represents a promising neuromodulatory approach. This prospective study aimed to assess the therapeutic effects of taVNS on functional recovery in this population.

**Methods:**

A total of 147 patients with post-stroke motor disorders were consecutively enrolled between February 2023 and November 2024. After excluding 8 dropouts, 139 patients were randomly assigned via a random number table to either an electrical stimulation group (taVNS group) or a rehabilitation group (conventional training). The taVNS group initially included 73 patients, with 3 dropouts yielding a final sample of 70. The rehabilitation group initially included 74 patients, with 5 dropouts resulting in 69 participants. All participants underwent comprehensive assessments at baseline and following a 4-week intervention period. Outcome measures encompassed neuroelectrophysiological parameters (motor evoked potential latency and amplitude), clinical functional evaluations (Action Research Arm Test, Fugl-Meyer Assessment for Upper Extremity, Modified Barthel Index), serum biomarker levels (brain-derived neurotrophic factor, S100 calcium-binding protein β), and systematic documentation of adverse events. Based on post-treatment Fugl-Meyer Assessment-Upper Extremity (FMA-UE) scores, patients were further categorized into improvement and non-improvement subgroups for additional comparative analysis. Pearson correlation analysis was utilized to examine potential relationships between functional scores, neurophysiological data, and biomarker concentrations.

**Results:**

Baseline characteristics were comparable between groups (*p* > 0.05). Post-intervention, the taVNS group showed significantly superior outcomes: shorter MEP latency (*p* < 0.05), higher MEP amplitude (*p* < 0.05), improved scores on ARAT, FMA-UE, and MBI (all *p* < 0.05), increased levels of BDNF (*p* < 0.05), and decreased levels of S100-β (*p* < 0.05). Within-group analysis indicated that MEP latency decreased only in the taVNS group, while amplitude improved in both groups. In the rehabilitation group, post-treatment MEP latency showed no significant difference from baseline (*p* > 0.05). Both groups exhibited significant post-treatment improvements in ARAT, FMA-UE, and MBI scores. However, the magnitude of improvement in clinical scores and biomarkers was substantially greater in the taVNS group after treatment. The pre-to post-treatment changes in MEP latency and MEP amplitude were larger in the taVNS group compared to the rehabilitation group (*p* < 0.001). Similarly, the changes in ARAT, FMA-UE, and MBI scores, as well as in BDNF and S100-βlevels, were all greater in the taVNS group than (*p* < 0.001). Adverse reaction incidence did not differ significantly between groups (taVNS 11.43% vs. rehabilitation 8.70%, *p* > 0.05). ARAT, FMA-UE, and MBI scores were negatively correlated with MEP latency and S100-β levels, and positively correlated with MEP amplitude and BDNF levels (all *p* < 0.05). These correlations were consistent for baseline values, post-treatment values, and pre-post change values.

**Conclusion:**

taVNS is an effective and safe adjunctive therapy for post-stroke motor recovery. It enhances neuroelectrophysiological function, improves motor and daily living abilities, and favorably modulates biomarkers of neural injury and repair. The consistent correlations among functional, neurophysiological, and biochemical outcomes highlight an integrated recovery pathway, supporting the integration of taVNS into standard neurorehabilitation protocols.

## Introduction

Stroke is one of the common clinical neurological diseases, imposing a heavy physical and psychological burden on patients and their families. Studies have shown that stroke is prone to cause permanent brain damage and has become a major cause of disability and death worldwide (1). According to epidemiological data, 80% of stroke patients may develop motor disorders of varying degrees after onset, mainly upper limb motor dysfunction. Due to the fine and complex nature of upper limb movements, which involve a large area of the central cortex, functional recovery is slow. This significantly affects patients’ activities of daily living (ADL), such as eating, dressing, and object retrieval (2). At the same time, long-term motor disorders can increase the risk of anxiety and depression in patients, directly delaying their rehabilitation process (2). Therefore, the adoption of effective early treatment measures can promote the rapid recovery of patients’ motor function and significantly improve their ADL.

At present, rehabilitation training serves as an important part of the rehabilitation treatment for patients with post-stroke motor disorders (3). Its goals not only focus on the recovery of motor function but also extend to the overall improvement of ADL, while taking into account the reconstruction of psychological and social functions (4). Previous studies have confirmed that rehabilitation training has a significant improvement effect on post-stroke motor disorders. It can not only effectively improve the walking speed, walking endurance, and balance function of stroke patients but also enhance their cardiopulmonary function and reduce cardiovascular risks (5, 6). However, in clinical practice, it has been found that although traditional rehabilitation training can improve some functions of patients with post-stroke motor disorders, it has certain limitations in regulating neural plasticity (7). After a stroke, the central nervous system of patients can be severely damaged; thus, the regeneration and remodeling of neurons are the keys to rehabilitation (8). Single rehabilitation training alone cannot directly stimulate the patient’s nervous system, making it difficult to effectively activate the regeneration of damaged neurons and the reconstruction of synapses. As a result, patients may achieve certain functional improvements during the rehabilitation process, but the recovery at the neural level progresses slowly, making it difficult to achieve the ideal rehabilitation effect (9). In recent years, with the improvement and development of medical technology, transcutaneous auricular vagus nerve stimulation (taVNS) has emerged as a promising non-invasive neuromodulation technique, which is primarily employed to facilitate the recovery of neurological functions. By selectively stimulating the auricular branch of the vagus nerve, taVNS activates the nucleus tractus solitarius and the dorsal motor nucleus in the medulla oblongata, thereby effectively improving the patient’s limb function (10). A previous study showed that taVNS combined with traditional rehabilitation training can significantly improve the motor function, sensory function, and emotional state of patients with acute stroke, with no obvious side effects (11). Another relevant study confirmed that taVNS, as an adjuvant treatment, can effectively promote the recovery of neurological function in patients with ischemic stroke and further improve the rehabilitation effect (12, 13).

However, there is currently no clear report focusing on the efficacy of taVNS in the treatment of patients with post-stroke motor disorders. This study was conducted to verify the efficacy of taVNS in patients with post-stroke motor disorders, thereby providing a new intervention method for the rehabilitation of post-stroke motor disorders and further promoting the development of rehabilitation medicine towards precision and individualization.

## Methods

### Study subjects

A prospective study was conducted. A total of 147 consecutive patients with post-stroke motor disorders admitted from February 2023 to November 2024 were included (8 patients dropped out, and 139 were finally included). Patients were assigned using a random number table to an electrical stimulation group (taVNS group) and a rehabilitation group. The taVNS group included 73 patients (3 dropped out, with 70 finally included) who received taVNS treatment, while the rehabilitation group consisted of 74 patients (5 dropped out, with 69 finally included) who received traditional rehabilitation training. Both groups received continuous symptomatic pharmacological treatment, and the intensity, duration, and frequency of rehabilitation training were kept consistent between groups. All patients and their family members were informed of the study details and signed the informed consent form.

### Randomization and blinding procedures

#### Randomization

A random number sequence was generated using SPSS 25.0, and group allocation was performed by a designated researcher according to the sequence. This procedure ensured allocation concealment and randomness, thereby minimizing selection bias.

#### Blinding procedures

This study adopted an assessor-blinded design. Examiners responsible for neuroelectrophysiological measurements, laboratory personnel, and scale assessors were all strictly blinded to group allocation. Prior to assessment, all evaluators received standardized training, and detailed, standardized operating procedures and scoring criteria were established. Laboratory staff analyzed outcomes based on coded samples, performing indicator measurements according to the codes to prevent group information from influencing the test results.

### Inclusion and exclusion criteria

#### Inclusion criteria

① All patients met the diagnostic criteria for stroke specified in the Chinese Guidelines for the Diagnosis and Treatment of Acute Ischemic Stroke (14). ② All patients had their first onset of stroke and received relevant treatment in this hospital. ③ Patients were 18–67 years of age. ④ The patients had complete clinical baseline data and follow-up information. ⑤ The patients had clear consciousness, no cognitive impairment, and could cooperate with the completion of relevant tests.

#### Exclusion criteria

① Poor compliance and failure to complete the prescribed course of relevant treatment. ② Presence of hematological diseases or malignant tumors. ③ Other neurological, muscular, or orthopedic diseases affecting motor function. ④ A history of traumatic brain injury. ⑤ Presence of skin lesions, infections, hyperalgesia, or intolerance to the stimulation area.

### General information

Clinical data of patients who met the inclusion and exclusion criteria were collected based on the hospital information management system. The data included gender, age, body mass index (BMI), smoking history, drinking history, educational level, monthly family income, history of hypertension, history of diabetes, type of stroke, affected side, and course of disease, and the National Institutes of Health Stroke Scale (NIHSS) (which consists of 15 items, with a total score ranging from 0 to 42, where higher scores indicate more severe neurological impairment) (15).

### Treatment methods

All patients received symptomatic drug treatment, including blood pressure reduction, cerebral cell nutrition, anticoagulation, and improvement of cerebral edema.

#### Rehabilitation group

Traditional rehabilitation training was adopted. Patients were guided to place their limbs in a proper position, turn over in bed, perform breathing exercises, joint activities, and sitting training in bed. For patients with immobile affected upper limbs, passive exercises were guided, and neuromuscular electrical stimulation was performed. For patients with active upper limb movement, muscle strength levels were scientifically evaluated, and targeted limb movement exercises were carried out. For patients without orthostatic hypotension, sitting balance exercises were guided to strengthen the core muscle strength of the trunk and conduct exercises for controlling the body in different directions, ensuring that patients achieved grade III sitting balance. Patients were also guided to perform daily living exercises, mainly including dressing, eating, washing, and feeding training. Each training session lasted 60 min, once a day.

#### taVNS group

On the basis of the same conventional rehabilitation training and symptomatic pharmacological treatment as the rehabilitation group, patients additionally received taVNS. A TENS-200A auricular vagus nerve electrical stimulator was selected, and the stimulation site was the left auricular concha area of the patient. Before treatment, alcohol cotton pads were used to routinely clean the electrodes and the ear treatment area. The earplug electrodes were properly fixed in the patient’s ear canal, and the auricle electrodes were closely attached to the auricular concha area. According to evidence-based recommendations in the field of stroke rehabilitation (16), the stimulation parameters were set as follows: an output pulse frequency of 20 Hz for 10 s alternating with 4 Hz for 5 s, with a pulse width of 0.3 ms. The stimulation intensity was adjusted to the maximum level tolerated by the patient, as jointly confirmed by two rehabilitation physicians. Each session lasted 60 min, 6 times per week, and both groups were treated for a total of 4 weeks.

### Detection of neuroelectrophysiological indicators

Neuroelectrophysiological indicators of the taVNS group and the rehabilitation group were detected before treatment and after 4 weeks of treatment. A magnetic stimulator (Yiruidi, Wuhan, Model CCY-I) was selected, and the motor evoked potential (MEP) function module was activated. Recording electrodes and reference electrodes were placed on the muscle belly and tendon of the abductor pollicis brevis muscle on the affected side of the patient, and the ground electrode was placed on the wrist of the affected side. Magnetic stimulation with 120% resting motor threshold was applied to the M1 area of the affected side of the brain. The MEP of the abductor pollicis brevis muscle on the affected side was recorded in detail. Five waveforms with high stability and large amplitude were selected to calculate the latency of MEP (time from the start of stimulation to the appearance of the recorded MEP signal, unit: ms) and the amplitude of MEP (peak height of the MEP waveform, unit: mV).

### Action Research Arm Test

Evaluations were conducted before treatment and after 4 weeks of treatment. This scale is an internationally accepted upper limb function test method, mainly used to evaluate the upper limb function of patients with cerebral cortex injury. It includes 19 test items, divided into four subscales: grasp, grip, pinch, and gross movement. Each test item is scored using a 4-level scoring method (0–3 points), where 0 points indicate inability to complete the action and 3 points indicate normal completion of the action. The total score for one upper limb ranges from 0 to 57 points, with higher scores indicating better function. The Cronbach’s *α* coefficient of this scale is 0.862, the test–retest reliability is 0.881, and the content validity is 0.904, showing good reliability and validity (17).

### Fugl-Meyer Assessment-Upper Extremity

Evaluations were performed before treatment and after 4 weeks of treatment. This scale is a subpart of the Fugl-Meyer Assessment, mainly used to evaluate the motor function of the upper limbs in stroke patients. It covers multiple aspects, such as reflex activities, motor function of the shoulder, elbow, wrist, and hand, as well as coordination and speed. There are 33 assessment items, and each item is scored according to the completion status. The total score is 66 points, with higher scores indicating better upper limb function. The Cronbach’s *α* coefficient of this scale is 0.842, the test–retest reliability is 0.913, and the content validity is 0.875, showing good reliability and validity (18).

### Modified Barthel Index

Evaluations were conducted before treatment and after 4 weeks of treatment. This scale is a tool used to evaluate patients’ ADL. It includes 10 items, such as eating, bathing, personal hygiene, dressing, bowel control, bladder control, using the toilet, going up and down stairs, transferring between bed and chair, walking on flat ground, and using a wheelchair. The total score is 100 points, with higher scores indicating stronger ADL. Based on the total score, patients can be classified into the following categories: normal (100 points), basically self-sufficient in daily life (60–99 points), moderate functional impairment (41–59 points), severe functional impairment (21–40 points), and completely dependent in daily life (≤20 points). The Cronbach’s *α* coefficient of this scale is 0.830, the test–retest reliability is 0.855, and the content validity is 0.892, showing good reliability and validity (19).

### Detection of nerve injury markers

Brain-derived neurotrophic factor (BDNF) and central nervous system-specific protein β (S100-β) in the taVNS group and the rehabilitation group were detected before treatment and after 4 weeks of treatment. Fasting venous blood (3 mL) was collected from the patients, centrifuged at 3000 r/min with a radius of 8 cm for 10 min to separate the serum. Detection was performed strictly in accordance with the requirements of the enzyme-linked immunosorbent assay (ELISA) method and the kit instructions (Shanghai Runyu Biotechnology Co., Ltd.).

### Safety evaluation

All patients were followed up, and the incidence of adverse reactions in the taVNS group and the rehabilitation group after 4 weeks of treatment was counted. The adverse reactions included limb swelling, shoulder pain, limb numbness, and limited movement.

### Observation indicators

Neuroelectrophysiological indicators, Action Research Arm Test (ARAT) scores, Fugl-Meyer Assessment-Upper Extremity (FMA-UE) scores, Modified Barthel Index (MBI) scores, BDNF levels, S100-β levels, and adverse reactions were compared between the taVNS group and the rehabilitation group before treatment and after 4 weeks of treatment. A Pearson correlation model was constructed to analyze the correlations between post-treatment upper limb function, ADL and MEP latency, MEP amplitude, BDNF, and S100-β in patients with post-stroke motor disorders.

### Statistical analysis

SPSS 25.0 statistical software was used for data processing and analysis. The normality of measurement data was tested by the Shapiro–Wilk test, and the homogeneity of variance was tested by the Levene test. Measurement data conforming to normal distribution and homogeneous variance were expressed as mean ± SD. Independent samples *t*-test was used for comparison between the two groups, and paired samples *t*-test was used for intragroup comparison. Measurement data not conforming to normal distribution were expressed as [M(P_25_, P_75_)], and the non-parametric Mann–Whitney *U* test was used. Count data were expressed as [*n* (%)], and *χ^2^* test was used. A *p*-value of <0.05 was considered statistically significant.

## Results

### Comparison of baseline data between the taVNS group and the rehabilitation group

The results showed that the average age of the taVNS group was 47.62, which was not significantly different from that of the rehabilitation group (50.12) (*p* > 0.05). The BMI of the taVNS group was 23.55, and that of the rehabilitation group was 23.97, with no significant difference between the two groups (*p* > 0.05). The monthly family income of the taVNS group was 6.87, and that of the rehabilitation group was 6.34, with no significant difference (*p* > 0.05). The course of disease in the taVNS group was 2.68, which was not significantly different from that in the rehabilitation group (2.98) (*p* > 0.05). Meanwhile, there were no significant differences between the taVNS group and the rehabilitation group in terms of gender, smoking history, drinking history, educational level, history of hypertension, history of diabetes, type of stroke, affected side and pre-treatment NIHSS and MBI scores (*p* > 0.05). These results indicate that the baseline levels of patients in each group were consistent (Table 1).

**Table 1:** Comparison of baseline data between the taVNS group and the rehabilitation group.

Baseline data		taVNS group (*n* = 70)	Rehabilitation group (*n* = 69)	*χ*^2^/*t*	*p*
Gender	Male	39	35	0.348	0.556
	Female	31	34		
Age ( $\bar{\chi }\pm s$ , years)		47.62 ± 15.83	50.12 ± 16.70	0.906	0.367
BMI ( $\bar{\chi }\pm s$ , kg/m^2^)		23.55 ± 1.64	23.97 ± 1.85	1.417	0.159
Smoking history	Yes	27	33	1.213	0.271
	No	43	36		
Drinking history	Yes	30	28	0.074	0.785
	No	40	41		
Educational level	Junior high school or below	25	32	1.633	0.201
	Senior high school or above	45	37		
Monthly family income ( $\bar{\chi }\pm s$ , thousand yuan)		6.87 ± 2.28	6.34 ± 2.11	1.422	0.157
History of hypertension	Yes	26	30	0.580	0.446
	No	44	39		
History of diabetes mellitus	Yes	23	26	0.354	0.552
	No	47	43		
Type of stroke	Cerebral hemorrhage	28	32	0.576	0.448
	Cerebral infarction	42	37		
Affected side	Left	36	29	1.233	0.267
	Right	34	40		
Course of disease ( $\bar{\chi }\pm s$ , month)		2.68 ± 0.85	2.98 ± 0.99	1.918	0.057
NIHSS scores		12.58 ± 4.03	11.87 ± 3.69	1.083	0.281
MBI scores		41.69 ± 13.89	42.04 ± 14.01	0.148	0.883

### Comparison of neuroelectrophysiological indicators between the taVNS group and the rehabilitation group

**Figure 1:**
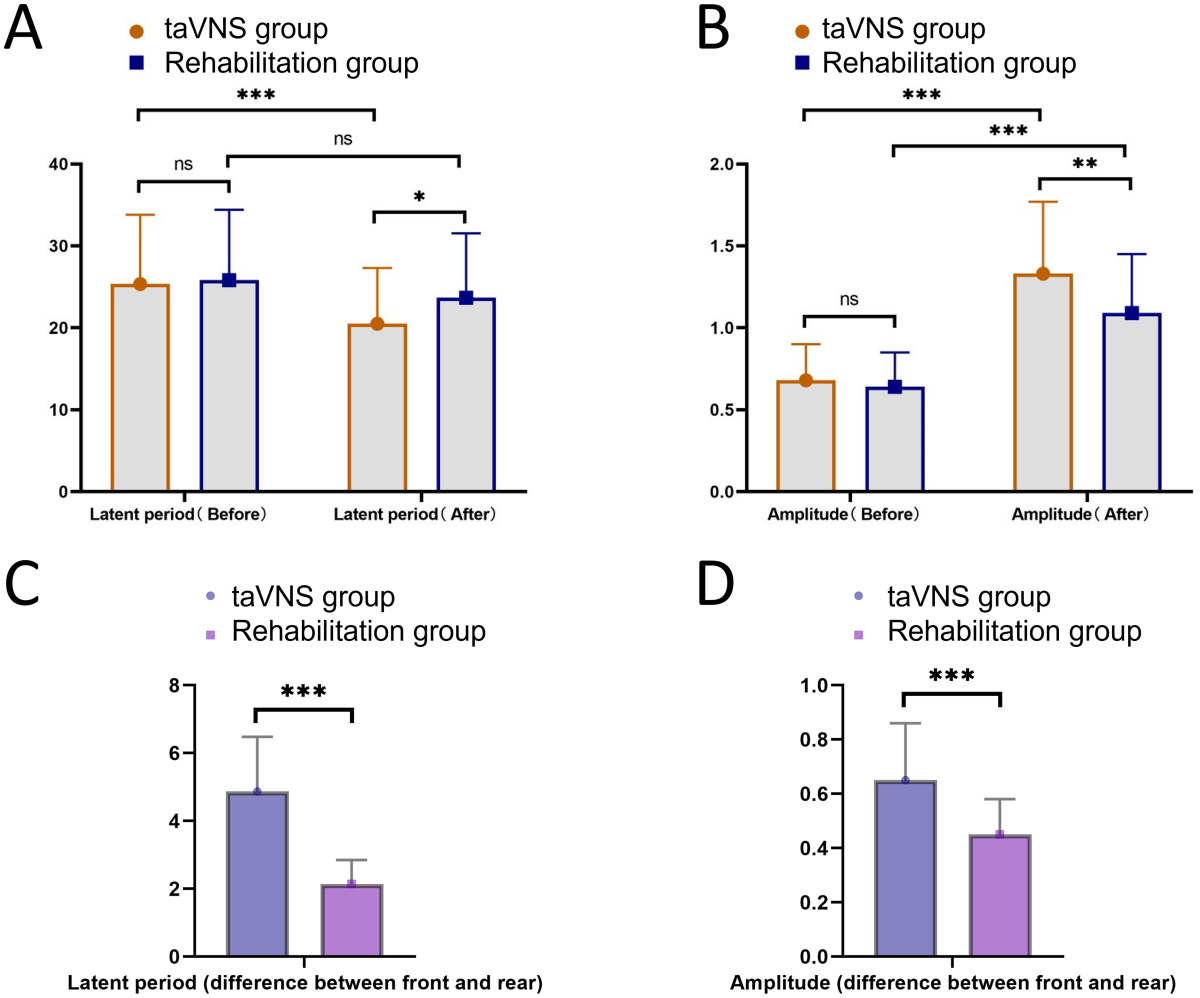
Comparison of neuroelectrophysiological indicators between the taVNS group and the rehabilitation group. **(A)** Comparison of latency between the two groups before and after treatment. **(B)** Comparison of amplitude between the two groups before and after treatment. **(C)** The between-group comparison of the pre-to-post-treatment change in latency. **(D)** The between-group comparison of the pre-to-post-treatment change in amplitude. ns indicates no statistically significant difference, * indicates *p* < 0.05, ** indicates *p* < 0.01, *** indicates *p* < 0.001.

Neuroelectrophysiological indicators are important tools for evaluating the neurological function status of patients. To illustrate the neuroelectrophysiological conditions of the taVNS and rehabilitation groups, the latency and amplitude of MEP were detected. The results showed that before treatment, MEP latency in the taVNS group was 25.36 ± 8.45 ms, which did not differ from that in the rehabilitation group (25.81 ± 8.60 ms; *p* = 0.756). After treatment, latency in the taVNS group decreased to 20.49 ± 6.82 ms, which was significantly lower than that in the rehabilitation group (23.67 ± 7.88 ms; *p* = 0.012). Within-group comparisons indicated that post-treatment latency in the taVNS group was significantly reduced compared to baseline (*p* < 0.001), whereas a slight decrease was observed in the rehabilitation group; however, it did not reach statistical significance (*p* = 0.130). Meanwhile, the pre-to-post-treatment change in latency was significantly greater in the taVNS group (4.87 ± 1.61) than in the rehabilitation group (2.15 ± 0.71; *p* < 0.001). Regarding MEP amplitude, no significant difference was found between the two groups before treatment (taVNS group: 0.68 ± 0.22 vs. the rehabilitation group: 0.64 ± 0.21, *p* = 0.275). After treatment, the amplitude increased to 1.33 ± 0.44 in the taVNS group, which was significantly higher than that in the rehabilitation group (1.09 ± 0.36; *p* = 0.001). Within-group analysis confirmed that amplitude significantly improved from baseline in both groups (both *p* < 0.001). Additionally, the pre-to-post-treatment change was significantly larger in the taVNS group (0.65 ± 0.21) than in the rehabilitation group (0.45 ± 0.13; *p* < 0.001, Figure 1).

### Comparison of motor function between the taVNS group and the rehabilitation group

**Figure 2:**
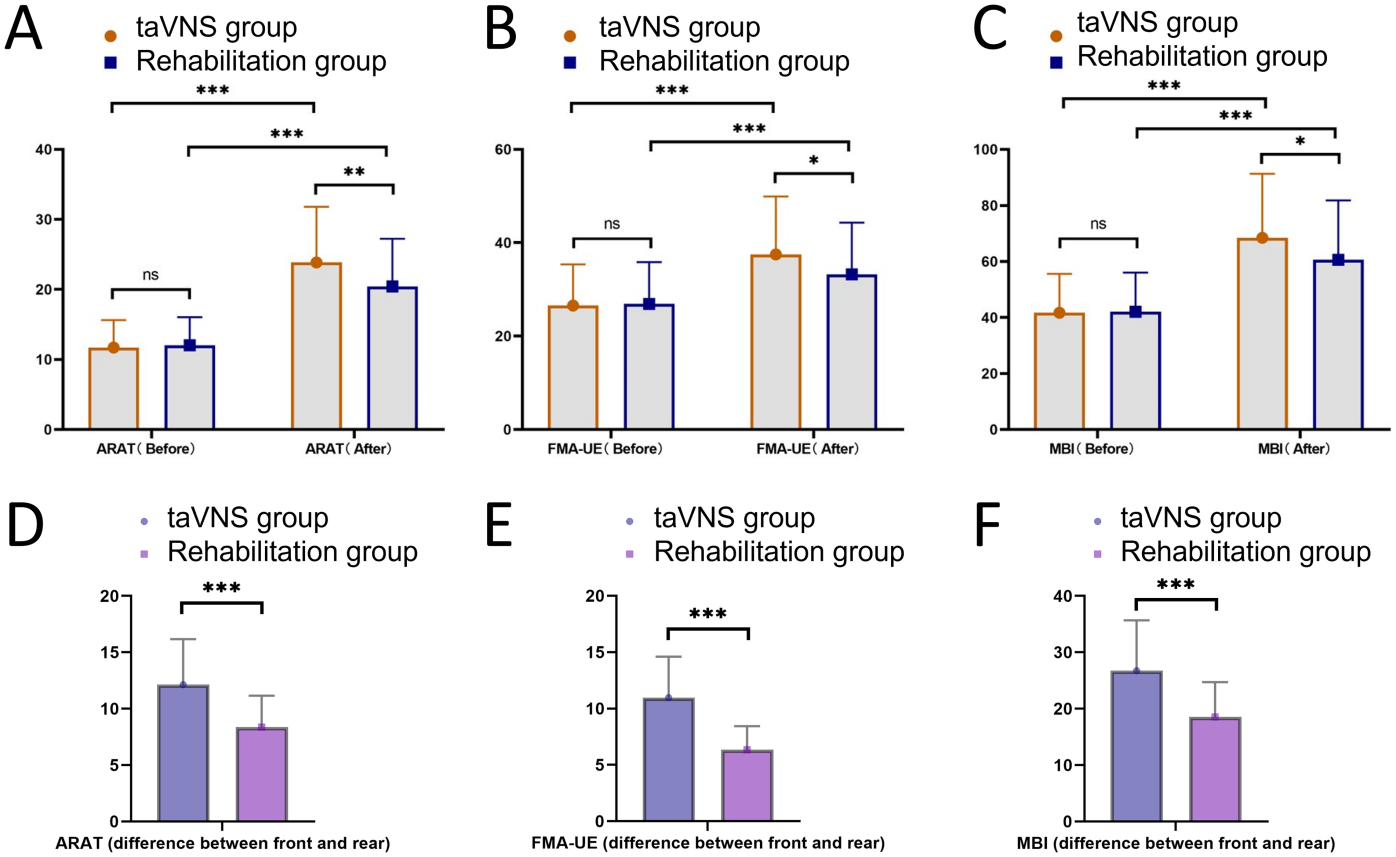
Comparison of relevant scores between the taVNS group and the rehabilitation group. **(A)** Comparison of ARAT scores between the two groups before and after treatment. **(B)** Comparison of FMA-UE scores between the two groups before and after treatment. **(C)** Comparison of MBI scores between the two groups before and after treatment. **(D)** The between-group comparison of the pre-to-post-treatment change in ARAT score. **(E)** The between-group comparison of the pre-to-post-treatment change in FMA-UE score. **(F)** The between-group comparison of the pre-to-post-treatment change in MBI score. ns indicates no statistically significant difference, * indicates *p* < 0.05, ** indicates *p* < 0.01, *** indicates *p* < 0.001.

The ARAT is a scale for evaluating patients’ upper limb and hand function, the FMA-UE is a scale for evaluating patients’ upper limb motor function, and the MBI is a scale for evaluating patients’ ADL. To illustrate the patients’ upper limb function and ADL, ARAT, FMA-UE, and MBI scores were measured. The results showed that before treatment, the ARAT score of the taVNS group was 11.72 ± 3.90, which was not significantly different from that of the rehabilitation group (12.05 ± 4.01, *p* = 0.624). However, after treatment, the ARAT score of the taVNS group increased to 23.86 ± 7.95, which was significantly higher than that of the rehabilitation group (20.43 ± 6.80, *p* = 0.007). Within-group comparisons showed that post-treatment ARAT scores were higher than pre-treatment scores in both groups (both *p* < 0.001). Meanwhile, the pre-to-post-treatment change was significantly greater in the taVNS group (12.13 ± 4.03) than in the rehabilitation group (8.38 ± 2.77; *p* < 0.001). At baseline, FMA-UE scores did not differ between the taVNS group (26.54 ± 8.83) and the rehabilitation group (26.91 ± 8.96; *p* = 0.807). After treatment, FMA-UE scores increased to 37.51 ± 12.40 in the taVNS group, which was significantly higher than in the rehabilitation group (33.25 ± 11.08; *p* = 0.035). Within-group comparisons analyses further indicated that FMA-UE scores improved significantly after treatment in both groups (both *p* < 0.001). Meanwhile, the pre-to-post-treatment change was significantly higher in the taVNS group (10.96 ± 3.64) than in the rehabilitation group (6.34 ± 2.10; *p* < 0.001). Similarly, baseline MBI scores were comparable between the taVNS group (41.69 ± 13.89) and the rehabilitation group (42.04 ± 14.01; *p* = 0.883). Following treatment, MBI scores increased to 68.45 ± 22.81 in the taVNS group, which was significantly higher than in the rehabilitation group (60.58 ± 21.19; *p* = 0.037). Within-group comparisons analyses also showed that post-treatment MBI scores were significantly higher than pre-treatment scores in both groups (both *p* < 0.001). Meanwhile, the pre-to-post-treatment change was significantly higher in the taVNS group (26.75 ± 8.90) than in the rehabilitation group (18.54 ± 6.17; *p* < 0.001, Figure 2).

### Comparison of nerve injury markers between the taVNS group and the rehabilitation group

**Figure 3:**
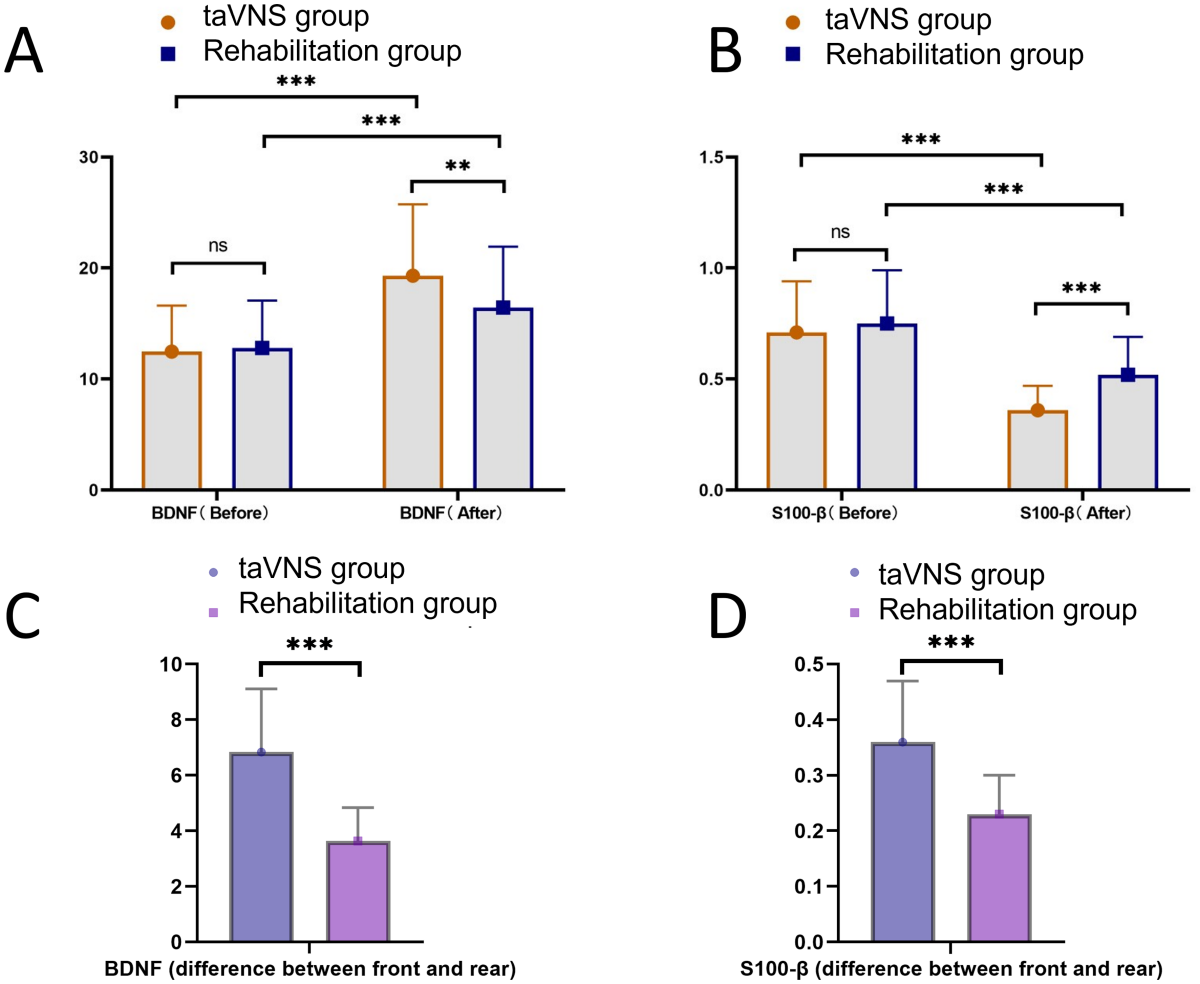
Comparison of BDNF and S100-β levels between the taVNS group and the rehabilitation group. **(A)** Comparison of BDNF between the two groups before and after treatment. **(B)** Comparison of S100-β between the two groups before and after treatment. **(C)** The between-group comparison of the pre-to-post-treatment change in BDNF. **(D)** The between-group comparison of the pre-to-post-treatment change in S100-β. ns indicates no statistically significant difference, ** indicates *p* < 0.01, *** indicates *p* < 0.001.

Nerve injury markers are used to evaluate the degree of nerve injury in patients. To illustrate the nerve injury status of the taVNS group and the rehabilitation group, BDNF and central nervous system-specific protein β (S100-β) were detected. The results showed that before treatment, the BDNF level of the taVNS group was 12.47 ± 4.15, with no significant difference from that of the rehabilitation group (12.81 ± 4.26) (*p* = 0.634); after treatment, the BDNF level of the taVNS group reached 19.31 ± 6.43, which was significantly higher than that of the rehabilitation group (16.45 ± 5.48) (*p* = 0.006). Within-group comparisons analyses showed that post-treatment BDNF levels were higher than pre-treatment levels in both the electrical stimulation and rehabilitation groups (both *p* < 0.001). Meanwhile, the pre-to-post-treatment change was significantly greater in the taVNS group (6.85 ± 2.27) than in the rehabilitation group (3.64 ± 1.20; *p* < 0.001). Before treatment, S100-β levels did not differ significantly between the taVNS group (0.71 ± 0.23) and the rehabilitation group (0.75 ± 0.24; *p* = 0.317). After treatment, S100-β levels decreased to 0.36 ± 0.11 in the taVNS group, which was significantly lower than in the rehabilitation group (0.52 ± 0.17; *p* < 0.001). Within-group comparisons analyses further indicated that S100-β levels were significantly reduced after treatment compared with baseline in both groups (both *p* < 0.001). Meanwhile, the pre-to-post-treatment change was significantly greater in the taVNS group (0.36 ± 0.11) than in the rehabilitation group (0.23 ± 0.07; *p* < 0.001, Figure 3).

### Comparison of the incidence of adverse reactions between the taVNS group and the rehabilitation group

To illustrate the safety of the taVNS group and the rehabilitation group, the incidence of adverse reactions in all patients after treatment was counted. The results showed that there were 8 cases of adverse reactions in the taVNS group, with an incidence rate of 11.43%, including 3 cases of limb swelling, 1 case of shoulder pain, 2 cases of limb numbness, and 2 cases of limited movement. In the rehabilitation group, there were 6 cases of adverse reactions, with an incidence rate of 8.70%, including 2 cases of limb swelling, 2 cases of shoulder pain, 1 case of limb numbness, and 1 case of limited movement. It can be seen that there was no significant difference in the incidence of adverse reactions between the taVNS group and the rehabilitation group (*p* > 0.05, Table 2).

**Table 2:** Comparison of the incidence of adverse reactions between the taVNS group and the rehabilitation $\bar{\chi }$ group.

Adverse reactions	taVNS group (*n* = 70)	Rehabilitation group (*n* = 69)	*χ* ^2^	*p*
Limb swelling	3	2		
Shoulder pain	1	2		
Limb numbness	2	1		
Limited movement	2	1		
Incidence	8 (11.43)	6 (8.70)	0.287	0.592

### Correlation between nerve injury status and motor and daily living abilities in patients

**Figure 4:**
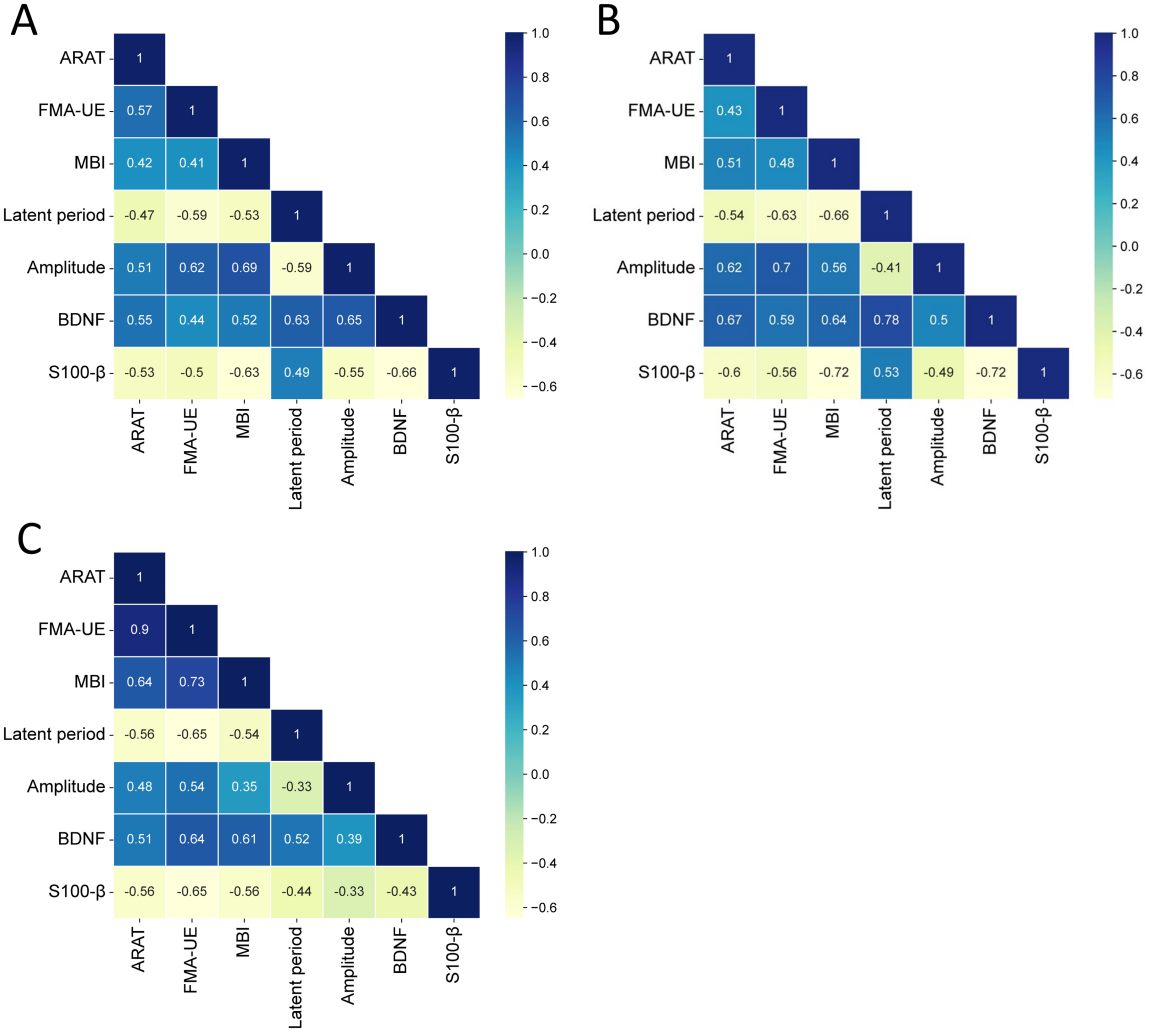
Correlation diagram between upper limb function, ADL and MEP latency, MEP amplitude, BDNF, S100-β in patients with post-stroke motor disorders. **(A)** The between-group comparison of baseline values. **(B)** The between-group comparison of post-treatment values. **(C)** The between-group comparison of the pre-to-post-treatment change.

A Pearson correlation model was constructed to analyze the correlations between post-treatment upper limb function, ADL at baseline and MEP latency, amplitude, BDNF, and S100-β in patients with post-stroke motor disorders. The results showed that ARAT was negatively correlated with MEP latency (*r* = −0.47, *p* < 0.05) and S100-β (*r* = −0.53, *p* < 0.05), and positively correlated with amplitude (*r* = 0.51, *p* < 0.05) and BDNF (*r* = 0.55, *p* < 0.05). FMA-UE was negatively correlated with MEP latency (*r* = −0.59, *p* < 0.05) and S100-β (*r* = −0.50, *p* < 0.05), and positively correlated with MEP amplitude (*r* = 0.62, *p* < 0.05) and BDNF (*r* = 0.44, *p* < 0.05). MBI was negatively correlated with MEP latency (*r* = −0.53, *p* < 0.05) and S100-β (*r* = −0.63, *p* < 0.05), and positively correlated with MEP amplitude (*r* = 0.69, *p* < 0.05) and BDNF (*r* = 0.52, *p* < 0.05). Post-intervention, ARAT was negatively correlated with MEP latency (*r* = −0.54, *p* < 0.05) and S100-β (*r* = −0.60, *p* < 0.05), and positively correlated with MEP amplitude (*r* = 0.62, *p* < 0.05) and BDNF (*r* = 0.67, *p* < 0.05). FMA-UE was negatively correlated with MEP latency (*r* = −0.63, *p* < 0.05) and S100-β (*r* = −0.56, *p* < 0.05), and positively correlated with MEP amplitude (*r* = 0.70, *p* < 0.05) and BDNF (*r* = 0.59, *p* < 0.05). MBI was negatively correlated with MEP latency (*r* = −0.66, *p* < 0.05) and S100-β (*r* = −0.72, *p* < 0.05), and positively correlated with MEP amplitude (*r* = 0.56, *p* < 0.05) and BDNF (*r* = 0.64, *p* < 0.05). Furthermore, the pre-post change in ARAT was negatively correlated with the pre-post change in MEP latency (*r* = −0.56, *p* < 0.05) and S100-β (*r* = −0.56, *p* < 0.05), and positively correlated with the pre-post change in MEP amplitude (*r* = 0.48, *p* < 0.05) and BDNF (*r* = 0.51, *p* < 0.05). The pre-post change in FMA-UE was negatively correlated with the pre-post change in MEP latency (*r* = −0.65, *p* < 0.05) and S100-β (*r* = −0.65, *p* < 0.05), and positively correlated with the pre-post change in MEP amplitude (*r* = 0.54, *p* < 0.05) and BDNF (*r* = 0.64, *p* < 0.05). The pre-post change in MBI was negatively correlated with the pre-post change in MEP latency (*r* = −0.54, *p* < 0.05) and S100-β (*r* = −0.56, *p* < 0.05), and positively correlated with the pre-post change in MEP amplitude (*r* = 0.35, *p* < 0.05) and BDNF (*r* = 0.61, *p* < 0.05, Figure 4).

## Discussion

Rehabilitation training is the main clinical treatment measure for patients with post-stroke motor disorders, which can especially improve the stability of patients’ proximal upper limb joints. However, it requires patients to persist in exercise for a long time and is easily affected by factors such as patients’ personal compliance, so its efficacy needs to be further improved (20). Therefore, the selection of safer and more effective treatment methods has become the focus of current clinical research. The latency and amplitude of MEP are important indicators for evaluating post-stroke motor disorders. Some studies have used MEP to see changes in cortical excitability in stroke patients after taVNS, finding that electrical stimulation can enhance excitability of the corticospinal tract and regulate the reorganization and modulation of functional connectivity networks in the brain through multi-pathway neuromodulatory mechanisms (21, 22). Another report indicates that the MEP latency of stroke patients may be shortened after effective treatment, suggesting the recovery of motor function (23). Consistent with these findings, the results of this study showed that after treatment, the MEP latency in the taVNS group was significantly lower than that in the rehabilitation group, while the amplitude was higher, indicating that taVNS can promote the recovery of patients’ neurological function. Analysis shows that MEP latency refers to the time from the stimulation of the motor cortex to the occurrence of motor response in the target muscle, which reflects the integrity of the central motor conduction pathway; while amplitude refers to the number and synchronization of excitatory neurons from the motor cortex to the target muscle. taVNS stimulates the auricular branch of the vagus nerve in patients and activates the nucleus tractus solitarius, thereby regulating the locus coeruleus noradrenergic system and the serotonergic system, and effectively enhancing neural plasticity (24). Noradrenaline and serotonin are key neurotransmitters that promote nerve repair and synaptic remodeling, and the increase in their levels is associated with the increase in MEP amplitude. In addition, taVNS can regulate vagal tone, reduce sympathetic excitability, effectively improve cerebral blood perfusion in patients, and provide a suitable metabolic environment for nerve repair. Among them, the recovery of autonomic nerve balance can promote the repair of the motor conduction pathway, which is manifested as the shortening of MEP latency (25). Based on this, the application of taVNS on the basis of traditional rehabilitation training can enhance the rehabilitation effect of patients and improve the latency and amplitude of MEP. It is noteworthy that randomization and blinded assessment were implemented in this study to reduce bias; however, potential confounding factors may still exist, such as individual variability, environmental influences, and measurement error, which could readily introduce bias into the results. Future studies should further control for these potential confounders to enhance the accuracy and robustness of the findings.

Metabolomic biomarkers play an important role in assessing post-stroke neuronal and brain injury, and can also elucidate the molecular mechanisms underlying post-stroke neural damage, thereby providing novel therapeutic targets for neurorehabilitation (26, 27). BDNF and S100-β are both widely used clinical biomarkers of neural injury that are involved in neuron survival, synaptic plasticity, and neurological function recovery. Among them, BDNF is a neurotrophic factor that is involved in the survival of neurons, synaptic plasticity, and the recovery of neurological functions. Once a stroke occurs, changes in BDNF levels are usually closely related to the recovery of neurological function and prognosis. S100-β is a calcium-binding protein. After a stroke, S100-β can be released from damaged cells into the blood and become an early biomarker of brain injury (28). A previous retrospective study found that rehabilitation training can promote the expression of BDNF in stroke patients, thereby promoting neuron repair and regeneration (29). Similar to this, the results of this study showed that after treatment, the BDNF level in the taVNS group was higher than that in the rehabilitation group, while the S100-β level was significantly lower, indicating that taVNS can improve patients’ neurological function and nerve injury status. Analysis reveals that Neuromodulation techniques play a positive role in neurorehabilitation. Compared with conventional rehabilitation training, neuromodulation approaches such as taVNS offer advantages including non-invasiveness and target specificity promotes the secretion of neurotransmitters, and acts directly on the cerebral cortex to enhance neuronal synaptic plasticity, thereby promoting the massive synthesis and release of BDNF and significantly increasing the BDNF level; at the same time, taVNS can activate α7 nicotinic acetylcholine receptors to inhibit the pro-inflammatory phenotype of microglia, reduce the production of inflammatory factors, and promote the expression of peroxisome proliferator-activated receptor γ, thereby up-regulating the BDNF level (30). In addition, taVNS can inhibit the excessive activation of microglia and astrocytes through the cholinergic anti-inflammatory pathway, reduce the pathological release of S100-β, and directly inhibit the expression of S100-β, resulting in a decrease in its level. Additionally, taVNS can modulate vagal tone, effectively reducing sympathetic excitability, enhancing cerebral blood flow perfusion, and creating a favorable metabolic environment for neural repair. The results of this study also showed that there was no significant difference in the incidence of adverse reactions between the taVNS group (11.43%) and the rehabilitation group (8.70%), suggesting that traditional rehabilitation training has been widely recognized for its safety through long-term clinical verification, and the addition of taVNS on this basis will not increase the incidence of adverse reactions. This is mainly because the current intensity, frequency, and duration of taVNS can all be adjusted according to the patient’s tolerance, and the vagus nerve endings are stimulated through ear electrodes without the need for implanted devices or surgical incisions, avoiding the adverse reactions that may be caused by traditional invasive vagus nerve stimulation. The combination with rehabilitation training does not introduce new risk factors; instead, it enhances the efficacy through a synergistic effect (31).

ARAT, FMA-UE, and MBI scores are all tools for evaluating the motor function and ADL of patients with post-stroke motor disorders. Stroke patients suffer from brain tissue damage due to cerebrovascular lesions, which directly impairs the central nervous system, especially the upper motor neurons and subcortical structures, thereby causing the loss of motor control ability and leading to motor disorders (32, 33). Previous studies have found that transcranial direct current stimulation, as a neuromodulatory approach, can promote motor recovery after stroke, particularly in improving upper limb motor function (32, 33). Additionally, it has been reported that one year after taVNS, the FMA-UE score increased by 9.2 points in stroke patients. In this study, taVNS was used to treat patients with post-stroke motor disorders. The results showed that after treatment, the ARAT, FMA-UE, and MBI scores in the taVNS group were higher than those in the rehabilitation group, indicating that taVNS has a significant effect on improving the upper limb function and ADL of patients with post-stroke motor disorders. taVNS can enhance the excitability of the motor cortex, improve the conduction function of the corticospinal tract, and promote the coordinated activation of the brain regions responsible for hand function, the basal ganglia, and the cerebellum, thereby enhancing patients’ fine motor abilities of the upper limbs, such as grasping, pinching, and manipulating objects (34). At the same time, taVNS can directly improve the efficiency of the motor conduction pathway by shortening the MEP latency and increasing the amplitude, enhancing the muscle control ability of the upper limbs, and further improving patients’ daily activity abilities such as dressing, eating, and washing (35). It can be seen that in addition to traditional rehabilitation training, the application of taVNS can not only improve patients’ limb function but also promote the improvement of their ADL. Relevant studies have shown that the FMA-UE score has good reliability, validity, and clinical application value in evaluating the improvement of post-stroke motor disorders (36, 37). In this study, a Pearson correlation model was constructed to analyze the correlations between post-treatment upper limb function, ADL and MEP latency, MEP amplitude, BDNF, and S100-β in patients with post-stroke motor disorders. The results showed that ARAT, FMA-UE, and MBI at baseline, post-intervention, and their pre-post changes were each negatively correlated with MEP latency and S100-β, and positively correlated with amplitude and BDNF. This indicates that there is a close internal relationship between the improvement of neuroelectrophysiological function (such as shortened latency, increased amplitude, decreased S100-β, and increased BDNF) and the recovery of patients’ physical functions (such as upper limb function, motor ability, and ADL). It suggests that in clinical practice, the improvement of patients’ upper limb function, motor ability, and ADL can be effectively promoted by improving neurological function and reducing nerve injury, which provides a theoretical basis for clinical treatment (38).

Nevertheless, this study still has certain limitations. For example, only patients with post-stroke motor disorders admitted to this hospital were included, and the sample size was small. Only the efficacy and safety of patients were evaluated 4 weeks after treatment, and no long-term follow-up was conducted. Although this study applied a standardized protocol for taVNS, the clinical heterogeneity of post-stroke motor impairment meant that certain individualized treatment variables could not be fully homogenized, and no stratified analyses were performed based on these variables, which may represent a potential source of confounding bias. In addition, the study focused on comparing the efficacy of taVNS with conventional rehabilitation training and relied solely on between-group and within-group comparisons without applying multiple-comparison corrections for the various outcome measures, thereby increasing the risk of inflated type I error and potentially weakening the credibility of differences observed in some secondary endpoints. Although the overall safety of taVNS is good, individual studies have mentioned adverse reactions such as hypotension and device-related discomfort, so long-term monitoring with a larger sample size is needed to clarify the risk–benefit ratio. In this study, the rehabilitation group received only conventional rehabilitation training, and no sham taVNS control group was established. As a result, it is difficult to fully exclude the influence of placebo effects, which may to some extent affect the precise determination of the specific therapeutic effects of taVNS. Therefore, in future clinical practice, patients with different subtypes of stroke should be included to conduct stratified analysis on the efficacy differences of taVNS in different populations; at the same time, a long-term follow-up database for stroke patients treated with taVNS should be established to collect clinical, imaging, and biomarker data, providing a basis for personalized prognosis prediction models; For multiple comparisons across numerous endpoints, a hierarchical adjustment strategy can be adopted according to outcome priority. For example, Bonferroni correction may be applied to core outcomes to control the overall type I error rate, whereas false discovery rate adjustment may be used for secondary outcomes to enhance statistical power while maintaining methodological rigor. A guideline for personalized stimulation parameters based on stroke type, lesion location, and inflammation level should be formulated, and the optimal scheme should be determined through dose-effect research. At the same time, a sham stimulation control group should be added to further clarify the true therapeutic value of taVNS.

In conclusion, taVNS has a good effect in the treatment of patients with post-stroke motor disorders. It can effectively regulate the levels of BDNF and S100-β, improve the neuroelectrophysiological characteristics of patients, promote the recovery of patients’ motor function, and has high safety. At the same time, the correlations between various indicators confirm that the improvement of neurological function is closely related to the recovery of limb function, which provides a scientific basis for clinical prognosis evaluation and personalized rehabilitation.

## Data Availability

The original contributions presented in the study are included in the article/supplementary material, further inquiries can be directed to the corresponding author.
